# Supplementary material for: Look out: an exploratory study assessing how gaze (eye angle and head angle) and gait speed are influenced by surface complexity
Source: PeerJ. 2020 Apr 3;8:e8838. doi: 10.7717/peerj.8838 (PMC7134013; doi:10.7717/peerj.8838)
Supplement: Supplemental Information 1 [file peerj-08-8838-s001.docx]

# Supplementary material

## S1. Eye tracker calibration

Before commencing walking, participants completed an eye calibration. The calibration tracked eye movements whilst the participants remained still at a distance of 320cm from a screen (173cm x 265cm), following recommendations made using the Arrington ViewPoint® manual (Arrington ViewPoint, 2010). The eye height of participants whilst standing was also measured.

## S2. Accuracy of inertia measurement unit sensors (IMUs)

Previous research has shown that IMUs can be used to calculate head yaw whilst walking outdoors [1]. However, the accuracy of IMUs to calculate head movement has not been tested. Furthermore, despite previous research showing IMUs to be accurate at calculating gait speed [2], this has not been tested for walking over irregular surfaces. We therefore tested the accuracy of IMU measures of head pitch angle and gait speed for flat and uneven surfaces. To achieve this, we compared data from IMUs to the gold standard assessment of spatiotemporal gait parameters, motion capture cameras (MOCAP).

S2.1 Method

The test involved one individual walking over the two indoor surfaces tested in the study (**Figure 1a** & **1d**). Two IMUs (Delsys TRIGNO™ IM, Boston, MA, USA) were placed on the body, positioned at the midline of the forehead and the lateral left shank. The lateral shank was selected following the methodology described by Li, Young [2], and this protocol was used for the gait speed calculation. MOCAP cameras (Qualisys Oqus 7 cameras) were used to record head pitch angle and gait speed using Qualisys Track Manager (version 2.15). Four reflective markers were placed on a headband strapped to the head and four markers were attached to a marker plate, placed at the lumbar region. Head pitch angle was calculated from the average vertical gyroscopic movement at the head and gait speed was calculated from the average acceleration and positional data of the four lumbar markers. .

The participant walked ten times over the indoor, smooth surface and the indoor uneven surface. To check the IMUs accuracy for gait speed calculation, the participant walked at a comfortable walking speed as well as a range between the fastest and slowest possible walking speeds.

S2.2 Accuracy of the IMUs to calculate head pitch angle and gait speed

Both head pitch angle and gait speed correlated highly between the MOCAP and the IMU data (0.98 and 0.89 for head pitch angle and gait speed respectively). For gait speed, the discrepancy between MOCAP and IMU results was greatest when walking fast over uneven surfaces. These speeds were faster than the fastest speeds of participants in the main study. For all other gait speeds, errors were similar to those obtained by Li, Young [2].

## S3. Validation of eye movement

We completed a validation study of the possible eye movement range in the vertical direction. 10 healthy adults (5 male, mean ± SD; age = 27.4 ± 1.1 years; height = 175 ± 9.2cm) were asked to rate their visual comfort across a range of eye angles (+40° to -70°) on a Likert scale between 1 and 5 (1 = “very fresh” 5 = “severe strain”. This scale was taken from Shibata, Kim [3]. Participants were instructed to keep their head still, whilst fixating at targets set incrementally (in steps of 10°) from their eye height (defined as 0°). Only eye movements between +30° and -50° were, on average, rated at a comfort rating of 4 (moderate strain) or below. Any recorded eye movements outside of this range were excluded from the analysis because participants were deemed unlikely to move their eyes to cause them severe strain.

## S4. Examples of time series data


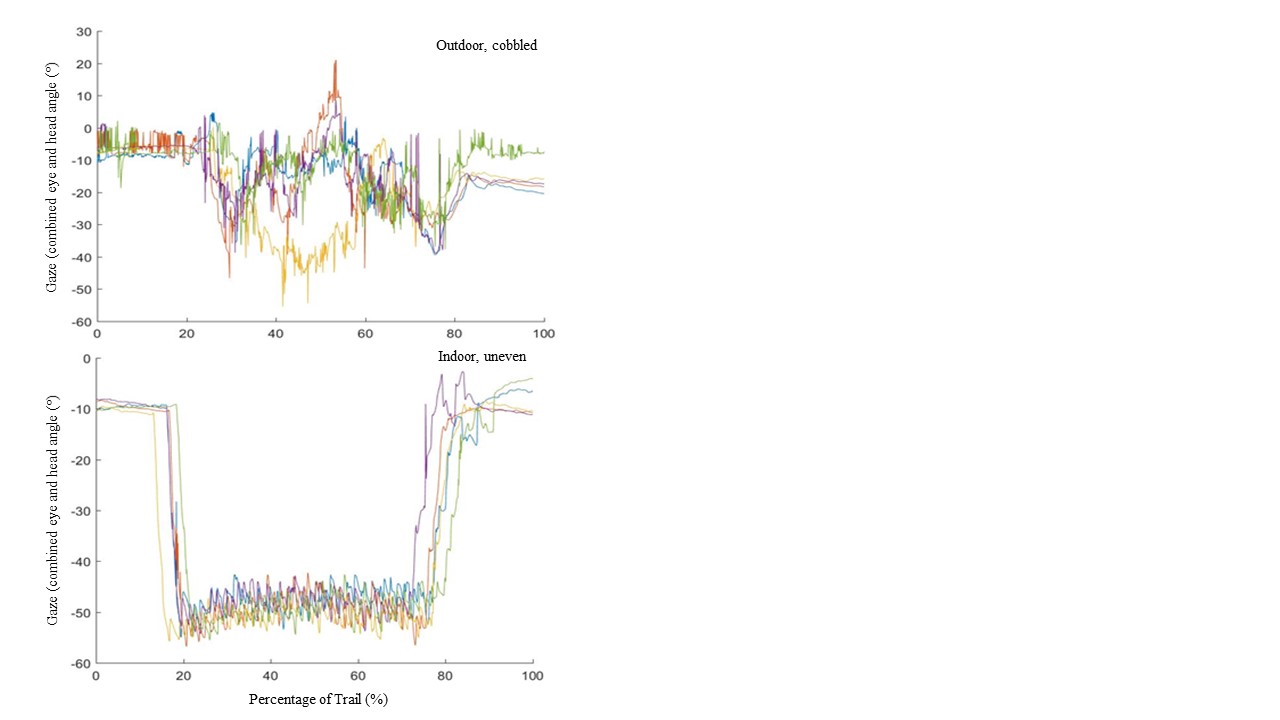


Sample gaze (combined eye and head pitch) angles (°) over the course of ten individual trials for one participant walking over the outdoor, cobbled surface (top) and the indoor, uneven surface (bottom). Different colours represent each of the ten trials in each of these two conditions.

[1] Tomasi M, Pundlik S, Bowers AR, Peli E, Luo G. Mobile gaze tracking system for outdoor walking behavioral studies. J Vision. 2016;16.

[2] Li Q, Young M, Naing V, Donelan JM. Walking speed estimation using a shank-mounted inertial measurement unit. J Biomech. 2010;43:1640-3.

[3] Shibata T, Kim J, Hoffman DM, Banks MS. The zone of comfort: Predicting visual discomfort with stereo displays. J Vision. 2011;11:11-.
